# Case of Splenic Leucocythæmia

**Published:** 1876-11

**Authors:** F. C. Curtis

**Affiliations:** Albany, N. Y.


### A CASE OF SPLENIC LEUCOCYTHÆMIA, EXHIBITING MARKED TEMPORARY IMPROVEMENT.

By F. C. CURTIS, M.D., Albany, N. Y.

The following case of an uncommon disease presents some unusual symptoms which render it of sufficient interest to be placed on record:

The patient, Daniel W., aged 39, a native of Albany, a fish-curer, with a family history clear with the exception of rheumatism, had an attack of rheumatism himself at the age of sixteen, and again another a year later. No heart complications were noted. With this exception he had always been healthy. At the age of eighteen he went West, and for four years was in the region of the Mississippi, up and down the river, much of the time in malarial districts, but, as far as he knew, never was affected with any malarial disease. His early life was rather irregular, but for a number of years he has been a sober, quiet, domestic man.

Four years ago he began to have night-sweats, which continued up to the advent of more urgent symptoms, within a year, with considerable constancy. Sweating took place almost every night, and was often very profuse. His skin became sallow, and he rather lost strength, although able to work until within a year of his death. He was of spare habit, and did not emaciate then or subsequently to any marked degree. His appetite was variable, but fair. He applied for relief of his night-sweats, and the usual round of remedies was tried, all having a temporary effect, and all failing eventually. There was no material change in his symptoms until a year previous to his death. Then he began to experience a dull, heavy feeling in the left hypochondriac region, increased by lying on the left side. Œdema of the feet also came on. In March, 1875, a protrusion of the cartilages of left lower ribs was noticed, and in May this grew so marked that he came to the office for examination. It was found that this side of the abdomen was occupied by a smooth mass, reaching fully to the median line at the umbilicus, the edge being sharply defined, and rounding off below into the pelvis, the upper limit being found by percussion to reach about to the sixth rib. The outline of the tumor was quite appreciable on inspection alone, the abdominal walls being thin. There was no tenderness on pressure over the mass, and its surface was perfectly smooth. The lymphatic glands were nowhere enlarged. Examination of the blood microscopically showed the number of white blood-corpuscles to be largely increased.

Various remedies were tried. In addition to a general tonic, quinia, which had been given previously for night-sweats, was resorted to in large doses; also in combination with arsenic; and, finally, iodide of potassium in five-grain doses in a bark and iron mixture, a mercurial plaster being applied over the enlarged organ. Under this last combination a slight diminution in the size of the spleen took place.

Early in July a new symptom set in; he began to experience dizziness, which lasted until he took to his bed. He walked with his legs apart, and with a groping gait, and had to use a cane to steady himself. When sitting down, and with everything quiet about him, this feeling entirely disappeared, but if objects about him were in motion, as when riding in a horse-car, he "felt as if everything was turning upside down."

He continued about until October, feeling fairly comfortable, somewhat asthenic, not much emaciated, and without marked cachetic appearance. There was a tendency to diarrhœa, especially after an acute attack of that disease in July. Dyspnœa was not noted. At the end of October he was taken with a profuse diarrhœa. He kept about for a week, and was then obliged to take to his bed, bronchitis and congestion of the lower portion of both lungs also coming on. No ordinary means served to check the diarrhœa, which averaged eight or ten semifluid stools per diem; and when the number of passages was diminished, he invariably felt worse. His pulmonary symptoms soon became urgent. Dyspnœa grew marked; auscultation showed congestion, and probably œdema of both lower lobes of the lungs. These urgent symptoms were relieved by direct treatment, in a measure. He was put upon the use of cod-liver oil, which he assimilated readily. Quinia and strychnia, with a bitter tonic, were also given. Iron, which was repeatedly tried, he could not bear. His symptoms all gradually improved, and toward the end of November the most remarkable fact connected with this case became apparent—the spleen began to rapidly diminish in size. Its edge, which reached a little beyond the median line at the umbilicus, receded till it was four inches to the left. It stopped at this point, and grew no smaller; neither did it again increase materially at any time, the edge being occasionally about half an inch nearer the median line; but care was necessary to put him in a uniform position, as the mass was movable, and could be lifted considerably to the right. The lower edge also came up out of the pelvis gradually and steadily. This diminution I am disposed to attribute mainly to the watery diarrhœa; perhaps in a measure also to the cod-liver oil, which was given not only as a nutrient, but with a view alo to the property that has been ascribed to it of increasing the number of the red blood-globules. His general condition gradually improved from the low state he was in during November, and he gained in strength so as to be able to sit up most of the day. His tongue was lightly coated, but was over-moist, almost dripping—or, if I should invent a suggestive term for this condition, which is not uncommonly met with, and which I have learned indicates a state of very poor physical reaction—I would call it a "dog's tongue." It is seen in the latter stages of organic diseases, and in some prolonged malarial fevers. The pulse was uniformly between 80 and 90; respiration 24.

After two attacks of venous thrombosis in December, he rallied very well, and made steady improvement in every respect, even as regards the size of the spleen, until the middle of January. But it is not my fortune to place on record the first case of recovery from leucocythæmia. He was taken, January 17, with chill and pain in the right side, with dyspnœa; and I found the pleura half filled with serum. This increased rapidly; orthopnœa and sleeplessness exhausted him completely, and he died ten days after the onset of the pleuritic attack.

An autopsy was made the day following his death. The right pleura was found filled with serum; lung flattened and collapsed. There were shreds of fresh lymph on the pleura. The pericardium was adherent to the heart. The right side of the heart was filled with clotted blood, which may be described as of a raspberry-chocolate color. The veins and venous cavities throughout the body showed this same curiously tinged, clotted blood, excepting some small veins which were filled with casts of a white color, and very firm, a knot of them having quite the appearance of a bunch of worms. The heart was enlarged; the mitral valves were thickened; the aortic valves adtheromatous, with points of calcification—spots of the same being also found on the aorta.

The spleen was adherent to the liver and other surrounding organs. Its capsule was thickened. It weighed about six pounds, and measured in its long diameter ten inches; transversely, seven and a half inches, and the thickness was five inches. As already mentioned, it had been much larger than this, but even such a magnitude is greater than occasionally reported. The notch was preserved; its tissue was hard and firm. Alcohol failed to preserve it, so unfortunately no microscopical examination was made.

The liver was also much enlarged, being eleven inches long, ten inches wide, and five and a half inches thick. It was apparently fatty.

The kidneys were large and white, having a thin cortical portion and a congested appearance of the pyramids, although the urine on examination during life was normal, except that it contained constantly the usual large amount of amorphous urates. There was no enlargement of any of the lymphatic glands.

The blood, about which the diagnosis of this case turns, was examined several times before death by myself and others. All specimens contained large colorless corpuscles in very greatly increased numbers. Dr. E. R. Hun said that, in a specimen which he examined, there were fields in which hardly a red globule could be seen. I am indebted to him for sending a specimen of the post-mortem blood to Dr. Joseph G. Richardson, of Philadelphia, who examined it with interest, and has very kindly reported to me the results of his investigations. He writes:

"The specimen of blood from your case, sent me by my friend, Dr. E. R. Hun, is very markedly leucocythæmic, the estimated proportion of white to red corpuscles being as 1 to 2, instead of 1 to 335 (the ratio given by Welcker in Strickea's Handbook). The method of enumeration I adopted was a plan of my own, although, I dare say, it may have previously occurred to other observers, viz.: simply to spread out the blood in very thin layers upon a slide, and allow it to dry; then, selecting suitable fields which were not too crowded, put on the cobweb micrometer eye-piece, and, adjusting the threads so as to cut off convenient spaces, count the red disks and white corpuscles respectively in each. By this plan any error from motion of the corpuscles is, of course, obviated.

"It may be worthy of note that the white globules in this specimen were unusually large, ten in a group which were measured averaging $\frac{1}{2193}$ of an inch, and one attaining the enormous magnitude of $\frac{1}{1786}$ of an inch. This peculiarity as to size of these corpuscles has been observed to sometimes occur by Prof. E. Wagner, who mentions it in his Manual of

General Pathology, page 546, American translation. Of course this immense enlargement may not have existed during life, and been merely connected with some post-mortem change in the *liquor sauguinis*, although my impression is that such was not the case."

In connection with this last statement, Dr. Richardson refers to an experiment, detailed on page five of his lecture on the Nature of the Salivary Globuels and their identity with the white blood Corpuscles, which showed that by diluting the serum of the blood by the aid of a filament of thread passing from a reservoir of fresh water to the covering glass under the microscope, a white corpuscle was seen to "gradually expand, displaying its delicate wall, with two rounded nuclei; then, after acquiring the magnitude of $\frac{1}{1700}$ of an inch, it exhibited the rapid and incessant movement of its contained molecules; and finally, when its diameter reached about $\frac{1}{1400}$ of an inch, it burst suddenly, discharging a portion of its contents."

In the specimens which I examined, taken during life, the proportion of white to red corpuscles was apparently much greater than that found by Dr. Richardson in the partly coagulated post-mortem blood, although no systematic counting was attempted.

I will do no more than conclude the simple detail of this case with a brief allusion to its more salient peculiarities. A very instructive analysis of a number of cases, by Dr. Edward G. Janeway, has not long since appeared in certain issues of the *New York Medical Record;* and I offer this as a contribution to the fund of cases from which such general facts are drawn.

A slight diminution in the size of the spleen followed the exhibition of iodide of potassium, but there was no history of syphilis.

Giddiness was a symptom peculiar to this patient, for I find no allusion to it by any author. It was very marked and persistent; not merely the result of anæmia, but evidently due to disturbance of brain function.

The rapid and very considerable diminution in size of the spleen is a noteworthy fact in this case. Occurring after very free diarrhœa suggests the idea that it was brought about in a measure thereby, especially as checking this was invariably followed by unpleasant symptoms.

Observation of this disease does not appear to have thrown much light upon the physiology of the spleen. The great objection to the theories both of Virchow and Bennett, that destruction of red blood disks or the manufacture of white ones in excess results from hypertrophy of this organ, exists in the fact that hypertrophy very frequently takes place without corresponding blood change. Spleenotomy, moreover, is done with impunity to dogs; and more or less complete removal of the spleen has been performed in man with success, M. Pean, in 1867, having removed the entire organ without ill result in a supposed case of ovarian tumor, which proved to be a large cyst of the spleen. These facts show that we have no definite knowledge of any important function of the spleen, and that we have no satisfactory data thus far whereby to determine the essential nature of a malady which, when of the splenic form, appears at first thought to be a functional disease of this organ.—*Ibid.*

